# Prevalence of pruritus associated with hemodialysis and its association with sleep quality among hemodialysis patients: a multicenter study

**DOI:** 10.1186/s12882-022-02838-z

**Published:** 2022-06-17

**Authors:** Mefleh Daraghmeh, Montaser Badran, Ahmad Janajreh, Mohanad Hassan, Adham Abu Taha, Amer A. Koni, Sa’ed H. Zyoud

**Affiliations:** 1 Department of Medicine, College of Medicine and Health Sciences, An-Najah National University, Nablus, 44839 Palestine; 2 Department of Nephrology, An-Najah National University Hospital, Nablus, 44839 State of Palestine; 3 Department of Biomedical Sciences, College of Medicine and Health Sciences, An-Najah National University, Nablus, 44839 Palestine; 4 Department of Pathology, An-Najah National University Hospital, Nablus, 44839 Palestine; 5 Division of Clinical Pharmacy, Department of Hematology and Oncology, An, Najah National University Hospital, Nablus, 44839 Palestine; 6 Department of Clinical and Community Pharmacy, College of Medicine and Health Sciences, An-Najah National University, Nablus, 44839 Palestine; 7 Poison Control and Drug Information Center (PCDIC), Faculty of Medicine and Health Sciences, An-Najah National University, Nablus, 44839 Palestine; 8 Clinical Research Center, An-Najah National University Hospital, Nablus, 44839 Palestine

**Keywords:** Prevalence, Pruritus, Sleep quality, Relationship, Hemodialysis, PSQI, 5-D itching score

## Abstract

**Background:**

CKD-associated pruritus (chronic kidney disease-associated pruritus) is one of the common symptoms in hemodialysis patients, with a major effect on sleep quality because it occurs at night. The main objective of this study is to determine the prevalence of pruritus among hemodialysis (HD) patients and its impact on sleep and investigate factors associated with pruritus and sleep quality.

**Methods:**

A cross-sectional study began in January until March of 2021 in HD centers of four different hospitals in the West Bank, Palestine. Patients with HD aged 18 years or older were included in our investigation. Pruritus and sleep problems were assessed by a 5-D itching score and the Pittsburgh Sleep Quality Index (PSQI) score.

**Results:**

Of 280 HD patients, 250 were accepted to participate in our study. The mean age of the participants was (54.9 ± 15.08). 62.8% were male, and 42.4% of the participants were elderly (age ≥ 60yrs). Pruritus was observed in 121 (48.4%). The 5-D itching score had a median [IQR] of 5.0[5.0–15.0], and 57.2% had a score ≥ 6 points. Severe pruritus was reported in 28.1% of patients. The score was significantly associated with residency (*p* = 0.033) and chronic comorbidities (*p* = 0.026). The PSQI score has a median [IQR] of 8[5–12], and 66.4% are poor sleepers with a score of < 5. The score was significantly associated with age (*p* = 0.017), marital status (*p* = 0.022), occupational status (*p* = 0.007), chronic comorbidities (*p* > 0.001), chronic medication (*p* = 0.008), severity of pruritus (*p* = 0.003) and duration of pruritus (*p* = 0.003). Regression analysis showed that the 5-D itching score and the total number of comorbidities were significantly associated with the PSQI score.

**Conclusions:**

Pruritus is a widespread complication among HD patients in Palestine. Pruritus also has major effects on sleep quality and is associated with poor sleep quality.

## Background

Pruritus is one of the main skin complaints and one of the most common skin problems. It is defined as an irritating skin sensation that can affect the quality of life [1]. Some studies show that there is a significant presence of people suffering from pruritus in the general population with a risk of 7% yearly of having it at least once in life. Moreover, there are many causes of pruritus worldwide classified as dermatological or nondermatological, such as systemic, psychiatric, neurological, or drug reactions. And, it is associated with them in different percentages, ranging from a quarter in hemodialysis (HD) patients to almost all patients with dermatologic diseases such as urticaria and atopic dermatitis (AD) [2].

The pathophysiology of CKD-associated pruritus (chronic kidney disease-associated pruritus) is still not known and is supposed to be a multifactorial problem depending on demographic, neuropathic, and psychogenic factors. During hemodialysis, many substances are produced from white blood cells, such as interleukin-1, which is released immediately after contact with a dialysis machine and membranes; interleukin-1 is thought to be one of the biological causes of pruritus [3].

CKD-associated pruritus is a prevalent and annoying complication that HD patients suffer from, which has a laborious effect on their quality of life. As it happens more frequently at night, it also affects sleep quality. Unfortunately, it is quite often ignored in hemodialysis patients, leading to a worsening of symptoms over time. Furthermore, HD patients who developed CKD-associated pruritus experienced delays in sleep and awakening. In 2019, a meta-analysis and systematic review revealed that the likelihood of sleep disturbances was 40% (95% CI = 0.30 to 0.49) [4].

The evaluation of pruritus using patient-reported outcomes is a priority for the patient, as there are no universally accepted biomarkers or objective measures of itch [5]. Similarly, evaluating sleep quality is equally important since patient-reported outcomes reflect the patient’s perception of their health and are associated with quality of life [6]. In fact, pre-dialysis and hemodialysis patients have significant burden symptoms (i.e., pain), which leads to poor sleep and reduced quality of life [7–9]. Furthermore, several previous analyses have shown that pruritus and insomnia are associated with poor quality of life [10–15]. Therefore, research to determine the prevalence and severity of pruritus and sleep is very important to guide future interventions.

Previous research conducted in various areas at the global level revealed that pruritus and sleep disturbances are detected in those who need dialysis routinely. However, the lack of data done in Palestine on this topic and the frustrating effects of pruritus on all aspects of the patients' life, physical side or psychological, make research on this topic important. Therefore, this study was designed to evaluate the prevalence of pruritus and sleep disturbance among dialysis patients and to determine the association of sociodemographic and clinical factors with pruritus, on the one hand, and the effect of these variables on sleep disturbance, on the other. It also explores the impact of pruritus on sleep quality. It is hoped that this research will contribute to a deeper understanding of pruritus and sleep disturbances among them, providing an important opportunity to find the best treatment to alleviate their suffering and grief.

## Methods

### Study design and setting

Our multicenter cross-sectional study was conducted in the HD centers in Tulkarem Governmental Hospital, Jenin Governmental Hospital, Tubas Turkish Hospital, and An-Najah National University Hospital from January to March 2021.

### Study population and sampling technique

We find that eleven dialysis centers serve HD patients from the West Bank population in Palestine as part of different hospitals. With a capacity of 1 to 50 dialysis machines. Our target was four of them located in the Northern West Bank. The total number of HD patients served by these centers was 714. The following equation is used to calculate the sample size in the prevalence study [16]; *n* = Z2*P (1-P) / d2, where *n* = sample to be calculated when the population size is greater than 10,000, z = 1.96 (CI 95%), d = 0.05 (absolute precision as a margin of error), *P* = 0.55 (the prevalence of pruritus from a pilot study of the same population). Therefore, the calculated sample size was 380 patients. As the population size is less than 10,000 (*N* = 714), we calculate the sample size using the adjusted sample formula = n/ (1 + (n/N)). We found that the minimum number of patients that we needed to include was 248. We used a margin of error of 5% and a confidence interval of 95%. We interviewed 280 patients using convenience sampling and received acceptance to participate in the study from 250 patients.

### Inclusion criteria

This article included patients 18 years of age or older who are currently on hemodialysis and do not have severe illnesses. Exclusion criteria were patients with cognitive impairment and those with incomplete or inconsistent data.

### Data collection instrument

We used a three-section questionnaire to interview our subjects based on some previously published studies [17–22]. Social and demographic data, including age, sex, level of education, occupation, level of monthly income, type of residence, and body mass index (BMI), were listed in the first part of the first section. The categories of BMI that we used were classified as the following: underweight (BMI < 18.5 kg/m^2^), normal (18.5–24.9 kg/m^2^), overweight (25–29.9 kg/m^2^), and obese (≥ 30 kg/m^2^); [18]. On the other hand, information such as the history of dialysis history (duration of HD in years), the duration of the session, the weekly frequency, the history of kidney transplantation, the number of chronically used medications and the total number of chronic comorbidities were defined in the second part of the first section [19].

The second part contains the 5-D itching scale in its validated Arabic version, which is a score used to evaluate pruritus, its intensity, duration and characteristics, and its effects on sleep, daily activities, leisure activities, and work. First, the intensities of pruritus were determined by asking the patient to categorize it into mild, moderate, severe, or unbearable categories. Then, the duration is classified into (less than 6 h, 6–12, 12–18, 18–23, and all day). After that, we asked about recent changes and their effect on sleep, daily activities, work, and social life. Finally, the locations where the patients felt pruritus. The level of itching ranges from 5 (no purities) to 25 (the most severe pruritus) [20]. Dr. Marlyn J. Mayo granted permission to use the scale via email.

The last section includes the Pittsburgh Sleep Quality Index (PSQI) test, which assesses sleep quality, daytime dysfunction, sleep duration, latency, habitual efficiency, disturbances, and medications to help sleep. This score consists of two parts, the first asking about bedtime, latency, waking up time, and duration in an open-ended type of question. The second part is answered by a number from 0 to 3, which demonstrates the incidence of sleeping trouble during the past month; if the patient answers 0 then it means that he or she has no trouble during the past month; with 1, having trouble once or twice a month; with 2, having trouble once or twice a week; and with 3 having trouble more than twice a week. The subject is counted as a poor sleeper if his total score is ≥ 5 [21]. The previously validated and translated Arabic version of the PSQI score was used with permission [22]. Fifteen participants participated in a pilot sample at the An-Najah National University Hospital to assess how comprehensible and understandable the questionnaire is. The PSQI was reliable and the internal consistency was adequate and good, with Cronbach’s α = 0.726.

### Ethical approval

Before starting our investigation, all aspects of the study protocol, including access to and use of patient clinical information, were approved by the Institutional Review Boards (IRB) and the Palestinian health authorities. Furthermore, informed oral consent was obtained from each patient after being informed of all the study objectives and required information.

### Statistical analysis

Data analysis was performed with the Statistical Package for the Social Sciences (IBM-SPSS) version 21. We describe the clinical, social, and demographic variables using descriptive analysis. The main variables (PSQI and 5-D itching score) were represented by mean ± SD or medians and interquartile ranges. Furthermore, their frequencies and percentages were included. The Mann–Whitney U test and the Kruskal–Wallis H test were our tools to test the associations between these variables, the PSQI score for sleep, and the 5-D itching score for pruritus. The association between PSQI and 5-D itching score was tested using the Spearman correlation. Subsequently, any variable with a p-value of less than 0.05, which was considered statistically significant, was entered into the multivariate analysis to find the predictors of PSQI and the 5-D itching score.

## Results

### Demographics of the study sample

Of the 280 HD patients interviewed in four hospitals in different cities, 250 were accepted to participate in our study analysis, representing an 89.3% response rate. The participants' social demographic, clinical, and dialysis characteristics are listed in full in Table 1. The mean age of the participants was 54.9 ± 15.08. 62.8% were men and 42.4% of the participants were elderly (≥ 60 years). About a third of the participants reached secondary school. The majority of participants (98%) came from low- and moderate-income families. In addition, a few percent of patients (20%) were employed. Our data showed that about 58.4% of the subjects lived on hemodialysis for less than or equal to 4 years. Furthermore, the three-session HD program a week was the predominant one by 93.2%. Approximately 30% of the participants lived with at least three total chronic comorbid diseases.

**Table 1:** Sociodemographic and clinical characteristics of the study sample (*n* = 250)

Variable	Frequency(%);*N* = 250
**Age categories(years)**	
18–30	25(10)
31–40	20(8)
41–50	37(14.8)
51–60	77(30.8)
61–70	59(23.6)
>70	32(12.8)
**Gender**	
Male	157(62.8)
Female	93(37.2)
**BMI category**	
Underweight	11(4.4)
Normal	86(34.4)
Overweight	80(32)
Obese	73(29.2)
**Education level**	
No education	35(14)
Primary	82(32.8)
Secondary	85(34)
High	21(8.4)
Graduated	27(10.8)
**Monthly income (NIS)**	
Less than 2000(low)	120(48)
2000–5000(moderate)	125(50)
5000–10,000(high)	5(2)
**Residency**	
Refugee camp	29(11.6)
Village	147(58.8)
Town	74(29.6)
**District**	
Nablus	101(40.4)
Tulkarm	64(25.6)
Jenin	57(22.8)
Tubas	28(11.2)
**Marital status**	
Single, divorced, and widowed	58(23.2)
Married	192(76.8)
**Living status**	
Alone	9(3.6)
With family	241(96.4)
**Occupation**	
Employed	50(20)
Unemployed	200(80)
**Dialysis vintage**	
**≤ **4 years	146(58.4)
> 4 years	104(41.6)
**Dialysis per week**	
**≤ **2 times	12(4.8)
3 Times	233(93.2)
4 Times	5(2)
**Dialysis session duration**	
3 h	90(36)
3.5 h	132(52.8)
4 h	28(11.2)
**Transplantation history**	
Yes	18(7.2)
No	232(92.8)
**Total chronic comorbid diseases**	
None	30(12)
1	80(32)
2	65(26)
3 or more	75(30)
**Chronic medications**	
<4	61(24.4)
≥4	189(75.6)

Abbreviations: BMI: Body mass index, NIS: new Israeli shekel (1 NIS = 0.30 US dollar)

### Characteristics of pruritus among HD patients

Among the 250 patients with HD, pruritus was observed in 121 (48.4%), with an overall mean ± SD of 5-D itching score was 9.37 ± 5.5 and a median [IQR] of 5.0 [5.0–15.0]. Most of the patients (53.7%) had a pruritus of fewer than 6 h per day and a small percentage (18.2%) suffered from it throughout the day. Regarding the severity of pruritus, a mild form was found in 23.1%, 29.8% with moderate pruritus, while severe and unbearable pruritus was reported in 28.1% and 19%, respectively. Most (68.6%) reported having trouble sleeping. In addition, they reported, with different percentages (56.2%, 54.5%, 45.4), an effect on their social life, daily activities, or work, respectively. The distribution of pruritus in the back, extremities, scalp, chest, and abdomen was reported (Table 2).

**Table 2:** Pruritus characteristics among HD patients with pruritus

Variable	Frequency(%);*N* = 121
**Pruritus severity**	
Mild	28(23.14)
Moderate	36(29.75)
Severe	34(28.1)
Unbearable	23(19.01)
**Duration of pruritus(hours)**	
<6	65(53.7)
6–12	19(15.7)
12–18	9(7.4)
18–23	6(5)
All the day	22(18.2)
**Number of patients with effect on**	
Sleep	83(68.6)
Social life	68(56.2)
Daily activities	66(54.5)
Work	55(45.4)
**Location of pruritus**	
Back	82(67.8)
legs	64(52.9)
Scalp	61(50.4)
Chest	53(43.8)
Arms	52(43)
shoulders	48(39.7)
Thighs	44(36.4)
Abdomen	43(35.5)
Palm of hand	30(24.8)
Tips of toes	28(23.1)
Buttocks	27(22.3)
Sole of feet	24(19.8)
Perineum	24(19.8)
Face	22(18.2)
Underclothes	13(10.7)

### Statistical analysis of 5-D itching score demographics

The most striking result to emerge from the data is that the 5-D itching score was significantly associated with residency (*p* = 0.033), where patients who resided in the refugee camp had the highest median score of 9[5–15.5]. Furthermore, the score was significantly associated with the total number of chronic comorbidities (*p* = 0.026). In the regression analysis, we reported that both factors (residency, *p* = 0.039 and comorbidities, *p* = 0.008) predicted the level of itching.

### Characteristics of sleep among HD patients

Sixty-six percent of the patients showed a poor PSQI response. According to Tables 3 and 4, the average sleep latency of the patients was 42 min and the average sleep duration was 5.7 h. The most common sleep disturbances were waking up at night or early in the morning (91.6%), difficulty getting to sleep (77.2%), nightmares (33.2%) and excessive daytime sleepiness (EDS) (49.2%). The most frequent causes of sleep disorders were snoring (43.6%), pain (45.2%), and breathing problems (45.6%). However, only 9.2% of them use medications to sleep.

**Table 3:** Characteristics of sleep in HD patients

Variable	Frequency(%);*N* = 250
**Time to go to bed (o'clock)**	
6–9	70(28)
9–12	163(65.2)
After 12 am	17(6.8)
**The time between going to bed and sleep (min)**	
<30	115(46)
30–60	97(38.8)
>60	38(15.2)
**Sleeping hours in the daytime(hours)**	
Don’t sleep	129(51.6)
1	62(24.8)
≥ 2	59(23.6)
**When they get up (am)**	
12–4	64(25.6)
4–8	160(64)
8–12	26(10.4)
**Sleeping hours at night**	
2–4	74(29.6)
5–8	155(62)
9–12	21(8.4)

**Table 4:** Problems that interrupt HD patients' sleep

	**Frequency(%);*N* = 250**			
Problem	**Not in last month**	**Once per month**	**Twice per week**	**Three or more times per week**
Not able to sleep within 30 min	57(22.8)	26(10.4)	43(17.2)	124(49.6)
Wake up at night or early morning	21(8.4)	19(7.6)	34(13.6)	176(70.4)
Going to bathroom at night	86(34.4)	23(9.2)	34(13.6)	107(42.8)
Inability to breath comfortably	136(54.4)	29(11.6)	49(19.6)	36(14.4)
Coughing or snoring loudly	141(56.4)	19(7.6)	34(13.6)	56(22.4)
Feeling very cold	117(46.8)	23(9.2)	39(15.6)	71(28.4)
Feeling very warm	182(72.8)	17(6.8)	26(10.4)	25(10)
Seeing bad dreams	167(66.8)	41(16.4)	19(7.6)	23(9.2)
Feeling pain	137(54.8)	18(7.2)	34(13.6)	61(24.4)
Need drugs to sleep	227(90.8)	9(3.6)	4(1.6)	10(4)
Having a problem to keep awakening during driving or eating	127(50.8)	26(10.4)	68(27.2)	29(11.6)

### Statistical analysis of PSQI demographics

The PSQI score has a median [IQR] of 8 [5–12] and 66.4% are poor sleepers, with a score of < 5. Furthermore, the PSQI score is significantly associated with multiple variables based on their demographic and clinical characteristics. These associations are as follows: age category (*p* = 0.017), marital status (*p* = 0.022), occupational status (*p* = 0.007), chronic co-morbidities (*p* > 0.001), chronic medications (*p* = 0.008), severity of pruritus (*p* = 0.003) and duration of pruritus (*p* = 0.003). Furthermore, the Spearman test showed a significant correlation between the PSQI score and the 5-D itching score (*r* = 0.235, *p* > 0.001).

The regression analysis showed that only the 5-D itching score (*p* = 0.022) and the total number of comorbidities (*p* = 0.001) predicted sleep quality.

## Discussion

This study aimed to investigate sleep quality and pruritus among HD patients and their relationship. It should be noted that a previous publication found that pruritus and poor sleep quality significantly affect survival in this population [23]. The current study contains important findings from a developing country regarding the commonness of pruritus in hemodialysis patients and its devastating effect on sleep. Certain country-specific variables that showed a significant association with pruritus or sleep give novelty to this study and insights to researchers to investigate this problem further and analyze these factors.

Pruritus is a troublesome problem for HD patients and should be appropriately managed. A recently developed treatment algorithm using topical medications for pruritus has shown to decrease the percentage of HD patients with pruritus and poor sleep related to this problem [24]. Our data found that almost half of HD patients have different grades of pruritus. The median score for 5-D itching was 5 (5–15), which is lower than a score from another study, 10.0 [8.0–12.0] [25]. Comparing our results with some other studies, we see in a study in Pakistan, conducted in 2019 that the prevalence of uremic pruritus among hemodialysis patients is high, 49.1% [26]. Moreover, we searched for another example in the Arab world and found one in Egypt, which found in 2014 that the prevalence of pruritus among HD patients was 51.6%.[27]. A lower percentage (37%) was reported in another study [28] and in a German analysis, which included 860 HD with a mean age of 67.2 years, and about half of them were men. The point prevalence of pruritus was 25.2%, the annual risk was 27.2%, and the lifetime risk was 35.2% [29]. In a meta-analysis, a study was conducted in China in 2018, with 11,800 patients and a total of 42 studies were included in this study. They found that the overall prevalence of pruritus among adult HD patients was 55% [30]. However, a higher percentage was reported in Malaysia (61.3%) and another Pakistani study (74%).

Our regression analysis showed that the 5-D itching score was significantly associated with residency and chronic comorbidities. Other factors related to residency may play a role in pruritus, such as lifestyle, environment, and types of food. However, unlike others, we did not find an association between female sex and pruritus [28].

We found that the sleep quality amongst HD patients is quite poor, with a PSQI > 5 in 66.4% of the patients. Our participants' median score for PSQI was 8 (5–12), which is similar to a study in Pakistan, 8.0 [7.0–10.0] [25]. Therefore, there is a similarity between the results we have come up with and many other previous studies [4, 30–35]. For example, 63% of HD patients were poor sleepers, as reported in a Turkish study in 2014 [34]. To talk about one more example, in 2018, 113 dialysis patients were included in a Pakistani study that used the tool that we interviewed our patients, such as our current study (PSQI) found that about 70% of the subjects were poor sleepers [35]. So, we can see that they are similar to our results.

The PSQI score was significantly associated with age category, marital status, occupational status, chronic comorbidities, chronic medications, the severity of pruritus and its duration, and there was a significant correlation between the participants’ rating of PSQI score and 5-D itching score (*r* = 0.235, *p* > 0.001). When comparing the results of our study with those of Pakistan, there was a significant correlation between pruritus and sleep score (*r* = 0.423, *p* < 0.001). Furthermore, the results revealed that there was an association between pruritus and the duration of CKD (*p* = 0.014) and the age of the patients (*p* = 0.038). In addition to a similar significant association between PSQI score and the pruritus and duration of CKD [25]. In Malaysia, 61.3% of the study participants reported having pruritus and most of them had mild pruritus. However, patients with more severe pruritus were found to have a higher incidence of experiencing poor sleep quality 5.47 times compared to others [3]**.**

Our findings are aligned with other studies conducted, which reported that CKD-associated pruritus is a difficult complication faced by patients receiving hemodialysis and affects their quality of life-related to health, such as sleep, social activities, housework, and work areas [12, 26, 36, 37]. Furthermore, it affects the quality of sleep and the disturbances that they may have. However, the variation in pruritus prevalence and sleep quality reported in our study and other studies may be related to differences in study design, study population and sample size, race, sampling technique, and definition of CKD-associated pruritus.

### Strengths and limitations

This study is the first on this subject in Palestine to investigate the association between pruritus and sleep quality among HD patients. Furthermore, being a multicenter study that covers patients from four centers in different regions of Palestine is also a strength.

This study has some limitations. First, this study used a cross-sectional design. As a result, causal conclusions may be impossible to establish. Second, we conducted these non-random sampling methods involving multicenter convenience sampling. Therefore, the generalizability of the research results has certain limitations. Finally, the current study did not cover or analyze certain factors, such as dialysis adequacy, laboratory results, residual kidney function, and the dialysis modality.

## Conclusions

In general, the findings of this study demonstrated that the prevalence of pruritus among HD patients is high and has a frustrating effect on sleep quality, and is associated with poor sleep quality. These findings suggest that we have a big problem, so we recommend a reasonable approach to address this issue, which could be a surveillance system among HD patients that helps us detect early such complications and an appropriate system that makes them more accessible to probable medications and treatment. Furthermore, we think that the findings of this study have important implications for future practice because it is a key policy priority to plan for the long-term care of those people.

## Data Availability

Data and materials used in this work are available from the corresponding author upon request.

## Abbreviations

IRB: Institutional Review Board
NIS: New Israeli shekel
DM: Diabetes Mellitus
SLE: Systemic Lupus Erythematous
HD: Hemodialysis
MI: Myocardial Infarction
CVA: Cerebrovascular Accident
HF: Heart Failure
COPD: Chronic Obstructive Pulmonary Disease
CKD-associated pruritus: Chronic kidney disease-associated pruritus
PSQI: Pittsburgh Sleep Quality Index
